# Primary Murine Myotubes as a Model for Investigating Muscular Dystrophy

**DOI:** 10.1155/2015/594751

**Published:** 2015-08-24

**Authors:** Natalia Smolina, Anna Kostareva, Joseph Bruton, Alexey Karpushev, Gunnar Sjoberg, Thomas Sejersen

**Affiliations:** ^1^Department of Women's and Children's Health, Karolinska University Hospital, Solna, 17176 Stockholm, Sweden; ^2^Center for Molecular Medicine, Karolinska University Hospital, Solna, 17176 Stockholm, Sweden; ^3^Federal Almazov Medical Research Centre, 2 Akkuratova Street, Saint Petersburg 197341, Russia; ^4^Institute of translational Medicine, ITMO University, 14 Birzjevaya Line, Saint Petersburg 199034, Russia; ^5^Department of Physiology and Pharmacology, Karolinska Institutet, 17177 Stockholm, Sweden

## Abstract

Muscular dystrophies caused by defects in various genes are often associated with impairment of calcium homeostasis. Studies of calcium currents are hampered because of the lack of a robust cellular model. Primary murine myotubes, formed upon satellite cell fusion, were examined for their utilization as a model of adult skeletal muscle. We enzymatically isolated satellite cells and induced them to differentiation to myotubes. Myotubes displayed morphological and physiological properties resembling adult muscle fibers. Desmin and myosin heavy chain immunoreactivity in the differentiated myotubes were similar to the mature muscle cross-striated pattern. The myotubes responded to electrical and chemical stimulations with sarcoplasmic reticulum calcium release. Presence of L-type calcium channels in the myotubes sarcolemma was confirmed via whole-cell patch-clamp technique. To assess the use of myotubes for studying functional mutation effects lentiviral transduction was applied. Satellite cells easily underwent transduction and were able to retain a positive expression of lentivirally encoded GFP up to and after the formation of myotubes, without changes in their physiological and morphological properties. Thus, we conclude that murine myotubes may serve as a fruitful cell model for investigating calcium homeostasis in muscular dystrophy and the effects of gene modifications can be assessed due to lentiviral transduction.

## 1. Introduction

Muscular dystrophies are a heterogenous group of genetic disorders characterized by muscle wasting and degeneration. Unraveling the pathogenesis of muscle dystrophies has great clinical and scientific importance and demands reliable cellular models for investigating underlying molecular mechanisms. Among various types of dystrophies Duchene muscular dystrophy (DMD) is well described due to availability of transgenic mice model, mdx mouse. These animals carry a point mutation in dystrophin gene, leading to appearance of premature stop codon which results in absence of full-length dystrophin [1]. It was shown that in murine model of DMD, mdx mouse intracellular calcium was twice greater than in wild type littermates. Calcium influx is increased since membrane is more permeable and cells undergo permanent calcium overload resulting in activation calcium dependent proteases [2]. Thus, calcium homeostasis is often hampered in muscular dystrophies, leading to enhanced proteolysis due to proteases activation by calcium ions [3]. Functional studies, especially assessment of calcium intracellular events, are of importance for clarifying molecular mechanisms underlying myodystrophies pathogenesis. However, data about calcium handling in muscular dystrophy were mostly obtained on single fibers isolated from mdx mice [4–6] or on primary myotubes formed from the mdx satellite cells [7]. Animal models are widely used as disease models; however, guided by 3R principles, the goal of scientists is to reduce animal usage in their studies and to rely on cell culture. The choice of relevant and informative cellular model is a key factor in successful analysis and dissection of signaling pathways in monogenic disorders. One of the major obstacles in skeletal muscle research is the lack of a good mature cell line model for studying neuromuscular disorders. A number of cell types have been traditionally used: primary mechanically [8–10] or enzymatically [11] isolated muscle fibers and satellite cells obtained from newborn animals and their subsequent differentiation and maturation into myotubes [12–15]. However, in the case of research attempting to identify the effects of mutations of calcium handling proteins, none of the hitherto used cell models is optimal.

Muscle fibers are terminally differentiated multinucleated cell that can be several centimetres long and are the basic repeating units of mature skeletal muscles. Primary isolated muscle fibres with tendons attached are the most reliable model for investigation of intracellular Ca^2+^ homeostasis and changes in muscle force production [8, 16]. However, due to the difficulty in isolating these cells in large numbers, the use of these cells in* in vitro* experiments is limited and researchers have resorted to enzymatically dissociated fibres to be able to monitor changes in Ca^2+^ homeostasis [17–19]. In an attempt to overcome this limitation, use has been made of satellite cells. These cells located between the sarcolemma and basal lamina are a potent pool of muscle progenitor cells that can proliferate and fuse to repair or even form new muscles fibers in response to injury or increased physical activity and thus provide some regenerative capacity to muscle [20–26]. Satellite cells can be isolated easily from skeletal muscle biopsies using various enzyme digestion protocols and have been used for up to eight to ten passages in culture [27–29]. Myotubes formed upon satellite cells fusion have been frequently used to examine cytosolic Ca^2+^ concentration ([Ca^2+^]_*i*_) at rest and in response to stimulation [12–15]. These studies utilized satellite cells obtained from newborn mice and rats, which makes the universality and applicability of this model questionable.

Investigation of the role of individual proteins effect is often carried out by transgenic means whereby a protein of interest is expressed in a modified form, temporarily knocked down, or overexpressed. One important aspect in the choice of a suitable muscle cell model for analysis of calcium homeostasis is the ease with which cells can be genetically modified via viral transduction. Several different types of viral transduction have been tried including adenoviruses, adenoassociated viruses, herpes simplex viruses, and lentiviruses. At this point in time, efficient genetic modification via viral transduction of primary adult muscle fibers is difficult. Limited data exists regarding the effective adenoassociated viral transduction of muscle fibers* in vivo* when viral suspension was injected intramuscularly [30].In another study high percentage of muscle fibres expressed reporter gene was achieved when 1.7 × 10^7^ transduction units of virus were applied for transduction; however, while protein expression from delivered virus was seen for several weeks, it was accompanied by a marked inflammatory and immune response [30]. When primary muscle fibers isolated from various muscle types were transduced via adenoviruses encoded *β*-Gal, the success rate 24 hours after transduction ranged from 64% (for animals at age two weeks) to 80% (for animals aged from one to three days). However, when the same approach was adopted in adult mice (six months), the efficiency of transduction dropped to 0% [31]. In contrast, the majority of satellite cells were amenable to adenoviral transduction regardless of the age of animal from which they were isolated. The level of expression of the introduced gene in the satellite cells was quite high (95% of cells expressed the *β*-Gal protein) [31].

Lentiviral (LV) transduction provides stable gene expression in postmitotic nondividing cells and is thus a promising tool for gene modification [32]. To date, positive transduction of muscle fibers was found only when virus was injected intramuscularly, that is,* in vivo* [33]. One other group has reported successful muscle fiber transduction via LV encoded *β*-Gal* in vitro*; however, these experiments utilized L6 myotubes and not adult muscle fibers [34]. There are reports confirming high efficiency of LV transduction for proliferating myoblasts, as well as myotubes, after 72 hours of differentiation [35].

In summary, an optimal muscle cell model for investigating molecular pathways underlying muscular dystrophy has to be morphologically and physiologically similar to mature muscle fibers and should undergo assessment of Ca^2+^ homeostasis. The goal of this study was to define a cell model of mature muscle cells that could be useful for studying Ca^2+^ homeostasis both with and without genetic modifications.

## 2. Materials and Methods

### 2.1. Muscle Fiber Isolation

Young (8–16 weeks old) C57BL/6 male mice were supplied by B&K Universal (Sollentuna, Sweden). All studies were approved by Stockholm North Local Animal Ethics Committee and Local Ethics Committee of Federal Almazov Medical Research Centre. Mice were sacrificed by cervical dislocation. Muscles were removed and placed in DMEM with 1% penicillin/streptomycin (Gibco, USA). Single muscle fibers were isolated from flexor digitorum brevis (FDB) muscle. Isolated muscles were cleaned of the connective tissue and tendons and placed in 2 mL of filtered 0.3% collagenase I (C0130, Sigma, Germany) dissolved in DMEM (Gibco, USA) supplemented with penicillin-streptomycin (Gibco, USA) for 2 h at 37°C. After digestion, muscles were washed with DMEM supplemented with 20% FCS (Gibco, USA) to remove the residual enzyme. Muscles were gently triturated in 2 mL of DMEM supplemented with 20% FCS. After trituration, fiber suspension was incubated for 10 min in plastic dishes, which was found to be optional to reduce the amount of nonmuscle cells contamination. After the 10 min incubation, the fiber suspension was plated on Geltrex-coated (Gibco, USA) glass bottom Petri dishes (P35G-0-20-C, Mattek, USA), 500 *μ*L of suspension per one dish. Geltrex was diluted in cold DMEM (1 : 100) and the glass bottom of the dishes were coated and incubated at 37°C for one hour after which the dish was washed with PBS several times to remove excess Geltrex. The fiber suspension was plated on the dish and left for 10 min to allow fibers to attach to the glass bottom before the addition of 2 mL of incubation media (DMEM supplemented with 20% FCS). The incubation media was renewed every two days by replacement of half of medium. Cells were cultured in an incubator at 37°C under a 5% CO_2_ atmosphere.

### 2.2. Primary Satellite Cell Isolation, Cultivation and Differentiation

Satellite cells were isolated via two strategies. In the first strategy, satellite cells were allowed to branch out of muscle fibers and attach to the dish bottom. In the second strategy, satellite cells were isolated as a “pure” culture by enzymatic dissociation of muscle fibers [28, 36, 37].

For the first strategy, muscle fibers were isolated from soleus and flexor digitorum brevis muscles by incubation in collagenase and subsequent trituration as described above and incubated until the satellite cells appeared in the dishes.

**Figure 1:**
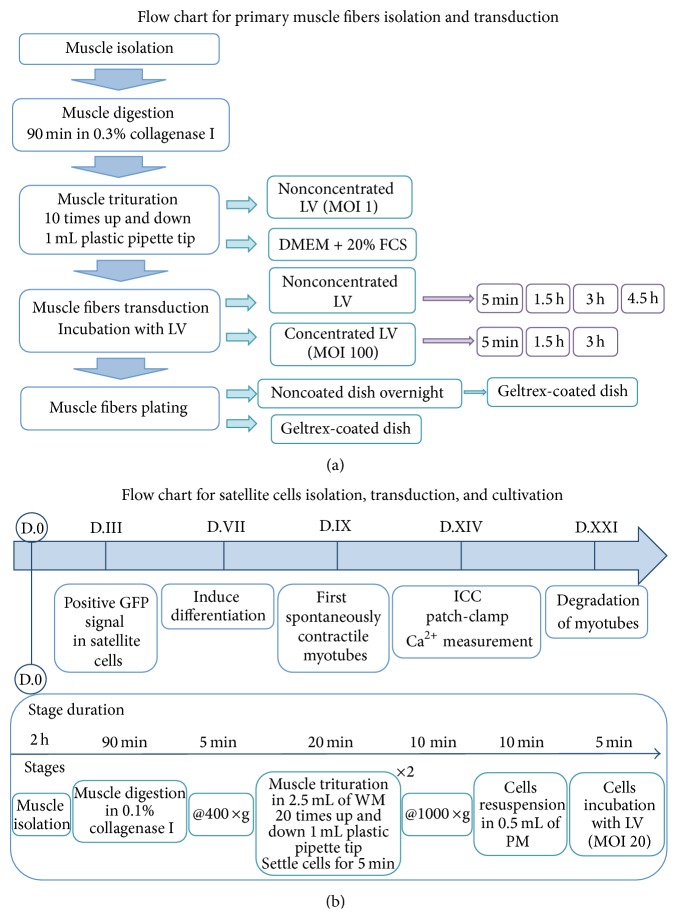
Flow chart for primary muscle fibers and satellite cells isolation and transduction. (a) Flow chart for primary muscle fibers transduction. Muscles after isolation underwent enzymatic digestion and then were triturated either in nonconcentrated LV or in DMEM supplemented with 20% FCS. After trituration in nonconcentrated LV (MOI 1), muscle fibers were transduced via nonconcentrated LV as well and were incubated with nonconcentrated LV for 5 min, 1.5 h, 3 h, or 4.5 h. Then muscle fibers were plated either directly to Geltrex-coated dish or cultivated on noncoated dish overnight and were then plated to Geltrex-coated dish. When cells were triturated in DMEM supplemented with 20% FCS, transduction was carried out either via nonconcentrated LV or via concentrated LV (MOI 100). Depending on LV type time of incubation varied, for nonconcentrated LV–5 min, 1.5 h, 3 h, and 4.5 h and for concentrated LV–5 min, 1.5 h, and 3 h. After transduction via concentrated LV cells were directly plated on Geltrex-coated dish. (b) Flow chart for satellite cells isolation, transduction, and cultivation. Satellite cells were isolated by means of enzymatic digestion and then centrifuged for 5 min at 400 ×g, and supernatant was discarded. Obtained cell pellet was twice resuspended in 2.5 mL of washing media (DMEM supplemented with 10% HS), suspension was settled for 5 min by gravity, and upper phase was transferred into fresh tube and spun down for 10 min at 1000 ×g. Cells pellet was dissolved in 0.5 mL of proliferation media (DMEM supplemented with 20% FCS, 10% HS, 1% CEE) and transduced via concentrated LV (MOI 20); polybrene at final concentration 8 *μ*g/mL was added to cells. 72 hours after transduction positively transduced cells were observed. Seven days after isolation cells reached confluence and were induced to differentiation. 48 hours after differentiation first spontaneously contractile myotubes were detected. After seven days of differentiation myotubes were taken in analysis (immunocytochemistry, patch-clamp, and calcium measurement). Three weeks after isolation myotubes started to degrade. LV, lentivirus; MOI, multiplicity of infection; PM, proliferation media; WM, washing media.

For the second strategy satellite cells were isolated enzymatically according to the protocol of Yablonka-Reuveni [38] with minor changes (Figure 1). In brief, isolated muscles were placed directly into enzyme solution, without any additional mechanical disruption with scissors. Digestion was done using collagenase type I instead of pronase. Muscle mincing was done using sterile blue pipette tips instead of glass Pasteur pipettes or serological pipettes; we did not filter the cell suspension through a strainer, since in our hands it decreased cell yields. The resultant satellite cells were plated on dishes coated with Geltrex instead of Matrigel. Thus soleus and FDB muscles were digested for 90 min at 37°C in 2 mL filtered 0.1% collagenase I (C0130, Sigma, Germany). To remove collagenase and cell debris after digestion, the cell suspension was centrifuged for 5 min at 400 ×g and the supernatant containing enzyme solution was discarded. To release satellite cells from the fibers the pellet was resuspended in 2.5 mL of washing media (DMEM supplemented with 10% horse serum (HS) (Gibco, USA)). After the resuspension the fibers were let to settle for 5 min and then the supernatant containing satellite cells was removed to a fresh tube. To increase satellite cells yield purity this step was repeated twice. The double-collected supernatant was centrifuged for 10 min at 1000 ×g, and the resultant supernatant was discarded and the pellet of cells was redissolved in 0.5 mL of proliferation media (DMEM supplemented with 20% FCS, 10% HS, and 1% chicken embryo extract (C3999, USBiological, USA)). Cells were plated on Geltrex-coated glass bottom petri dishes and cultured in proliferation medium until 80% confluence was reached. Fusion of some cells without external stimuli (differentiation media) was observed usually after 7 days of cultivation and served as a reliable indicator after which we induced differentiation. To induce satellite cell differentiation, the proliferation media was removed, cells were washed once with prewarmed PBS, and then differentiation media was added (DMEM supplemented with 2% HS). The differentiation media was renewed every other day by replacement of half of medium. Cells were cultured in an incubator at 37°C under a 5% CO_2_ atmosphere.

### 2.3. Lentiviruses Production and Cell Transduction

The pLVTHM (20 *μ*g), pMD2G (5 *μ*g), and packaging pCMV-dR8.74psPAX2 (5 *μ*g) plasmids were cotransfected into HEK-293T cells by a calcium phosphate method. The resultant production of lentivirus was concentrated by an ultracentrifugation method (20000 ×g for 2 h at 4°C), resuspended in 1% BSA, frozen in aliquots at −80°C, and titered using HEK-293T cells as described previously [39] (http://tronolab.epfl.ch/).

Several different approaches were tested to successfully transduce primary muscle fibres. To facilitate transduction, polybrene (Sigma, Germany) at a final concentration 8 *μ*g/mL was added to all transduced cells. We used (i) nonconcentrated virus and DMEM supplemented with 20% FCS as solution for muscle trituration and (ii) nonconcentrated and concentrated viral suspension as transduction agent and varied (iii) the incubation time with viruses and (iv) the type of plating surface (Figure 1). For transduction of the satellite cells, concentrated viral suspension at multiplicity of infection of 20 was added to the cells and incubated for 5 min before plating. Sixteen hours after transduction, the culture medium was completely replaced with fresh medium. To assess efficiency of viral transduction viruses coding for GFP were used in parallel.

### 2.4. Immunocytochemistry

The myogenic nature of the isolated cells was confirmed by immunocytochemical staining. Cells were fixed in 4% paraformaldehyde for 10 min at 4°C and then permeabilized with 0.05% Triton X-100 for 5 min. Nonspecific binding was blocked by incubation of permeabilized cells in 15% FCS for 30 min. Cells were incubated for one hour at room temperature with the following primary antibodies: anti-desmin (D33, DAKO, Denmark), anti-myosin heavy chain (MAB4470, R&D, USA), anti-ryanodine Receptor 1 (D4E1, Cell signaling, USA), anti-Mitofusin 2 (ab56889, Abcam, USA), anti-lamin A/C (NCL-LAM-A/C, Novocastra, UK). The secondary antibodies conjugated with Alexa Fluor 546 (Molecular Probes, USA) were applied for 45 min at room temperature. Nuclei were counterstained with DAPI (Molecular Probes, USA).

### 2.5. Whole-Cell Patch-Clamp

Ca^2+^ current was recorded in muscle fibers and myotubes using the whole-cell patch-clamp technique. Current recordings were performed with an Axopatch 200B amplifier and Digidata 1440A AD/DA converter (Molecular Device, USA). Data collection and analysis were done using pClamp 10.2 (Molecular Device, USA). Patch pipettes (1.5–4 MΩ) were pulled from borosilicate glass capillaries (World Precision Instruments, USA) by means of a micropipette puller P-1000 (Sutter Instruments, USA). The pipette solution had the following composition (mM): 120 CsCl, 5 MgАТP, 10 EGTA, and 10 HEPES (adjusted to рН 7.4 using CsOH) and the bath solution contained the following (mM): 120 ТЕА-Cl, 10 CsCl, 1.8 CaCl_2_, 1 MgCl_2_, 10 HEPES, 0.001 ТТХ, and 10 glucose, (adjusted to рН 7.4 using ТЕА-OH). Ca^2+^ current was evoked with a series of 200 ms depolarizing steps from −30 to 40 mV with 10 mV increments. In order to compare Ca^2+^ currents in different cells, Ca^2+^ current was normalized to the membrane capacitance.

### 2.6. Loading Cells with Calcium Indicators

Free intracellular Ca^2+^ was measured using the nonratiometric calcium indicator fluo-3 AM (Molecular Probes, USA). Rhod-2 AM (Molecular Probes, USA) was used to monitor free calcium in the mitochondrial matrix. Cells were incubated for 30 min with 2 *μ*M fluo-3 AM or 5 *μ*M rhod-2 AM and then washed for 20 min with Tyrode buffer at room temperature.

### 2.7. Stimulation of Sarcoplasmic Reticulum Ca^2+^ Release and Laser Confocal Microscopy

Cells were stimulated chemically with 2 mM 2-chloro-*m*-cresol (CmC, Sigma, Germany) or electrically at 1 Hz, 10 Hz, or 100 Hz.

A BioRad MRC 1024 unit (BioRad Microscopy Division, Hertfordshire, England) with a dual Calypso laser (Cobolt, Solna, Sweden) mounted on a Nikon Diaphot 200 inverted microscope was used. In the majority of experiments, a Nikon Plan Apo 20x dry lens (N.A. 0.75) was used. The fluo-3 AM was excited with 491 nm light and emitted signal was collected at 515 nm, the rhod-2 AM was excited with 532 nm light and the emitted light collected through a 585 nm long-pass filter. Confocal images were captured every 7 s and a total of 42 images were obtained for every experimental condition.

## 3. Results

### 3.1. Muscle Fiber Isolation and Transduction

**Figure 2:**
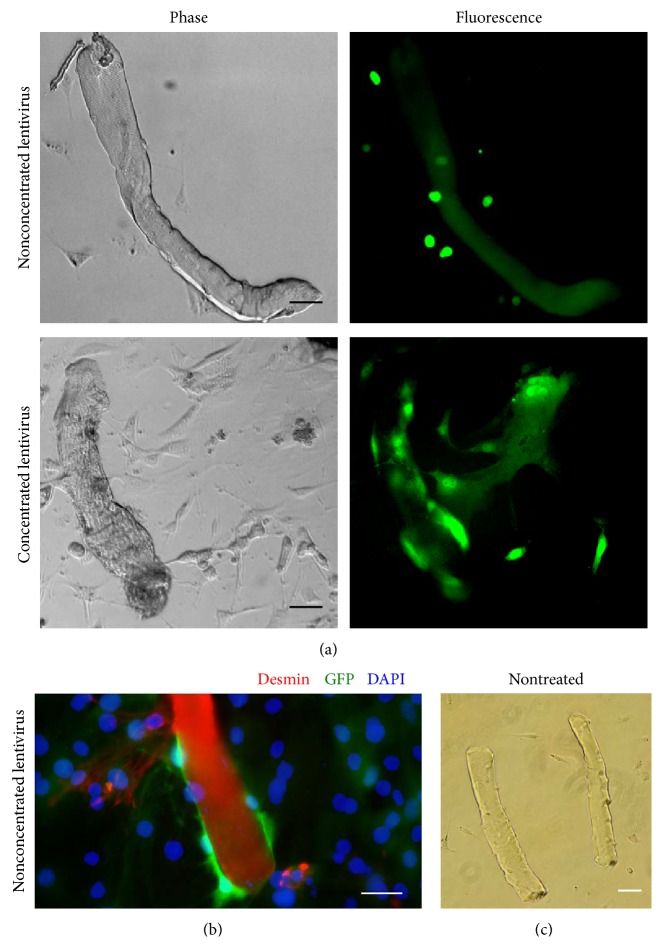
Muscle fiber transduction via lentiviruses. (a) In 72 h after muscle fibers transduction via nonconcentrated LV encoded LMNA (1 hour incubation with LV) muscle fibres kept their cross-striated pattern (upper panel). When concentrated LV encoded GFP was applied muscle fibers acquired positive signal, however, lost their cross-striation (lower panel). Satellite cells in both applications were positively transduced. (b) In 72 h after muscle fibers transduction via nonconcentrated LV (1 hour incubation with LV) muscle fibres did not express GFP, while it expressed desmin (red), however, branching out satellite cells expressed GFP. (c) Nontreated muscle fibers retain their cross-striation pattern and did not differ from muscle fibers transduced via nonconcentrated LV. Scale bar corresponds to 50 *μ*m.

**Figure 3:**
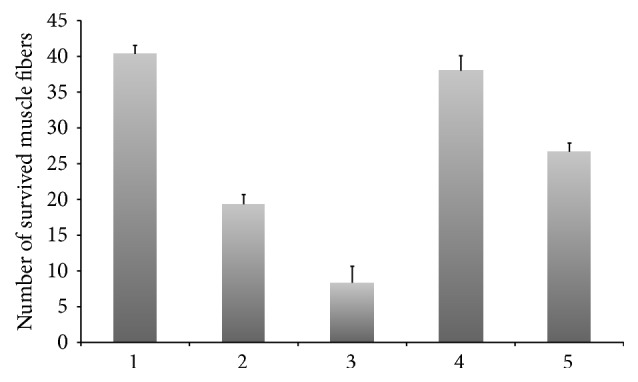
Graph showing numbers of living muscle fibers depending on type of isolation. 1: trituration in nonconcentrated LV, incubation with LV–5 min, and plating direct on Geltrex-coated dish; 2: trituration in nonconcentrated LV, incubation with LV–3 h, and plating direct on Geltrex-coated dish; 3: trituration in nonconcentrated LV, incubation with LV–3 h, overnight preplating on non-coated dish, and then plating on Geltrex-coated dish; 4: trituration in DMEM supplemented with 20% FCS, incubation with concentrated LV–5 min, and plating direct on Geltrex-coated dish; 5: trituration in DMEM supplemented with 20% FCS, incubation with concentrated LV–3 h, and plating direct on Geltrex-coated dish (*n* = 3 dishes).

Dissociated flexor digitorum brevis muscle fibers demonstrated a cross-striated pattern and contracted in response to electrical stimulation in the same way as mechanically dissected muscle fibers; that is, a larger transient increase of fluo-3 was observed upon increasing the stimulation frequency from 1 to 10 to 100 Hz. However, we were not able to obtain effective positive transduction of these primary muscle fibers. Using nonconcentrated virus, no GFP signal (to confirm that transfection has occured successfully) was detected in the muscle fibers, although satellite cells branching off the muscle fibers expressed GFP (Figures 2(a) and 2(b)). Increasing the incubation time from 5 minutes to 90 minutes, 3 hours, and 4.5 hours with nonconcentrated viruses resulted in a reduced number of living muscle fibers. For example, in one experiment, the number of living muscle fibers plated immediately after isolation was twice as great as the number alive after 3 hours or 4.5 hours of incubation in the nonconcentrated viral media (40 and 20 living muscle fibers, resp.) and four times greater than after overnight incubation on noncoated dish (10 living muscle fibers) for both muscle fibers and satellite cells quantity (Figure 3). Similar results were seen when the experiment was repeated on two other occasions. When concentrated virus was used for transduction, GFP signal was observed in both muscle fibers and in satellite cells 72 hours after transduction (Figure 2(b)). However, positively transduced muscle fibers were unable to survive in culture for longer than 24 hours, lost their cross-striated pattern and did not contract in response to electrical stimulation. In contrast, fibers that were not exposed to LV retained their morphological appearance and physiological response to electrical stimulation during 72 hours of observation. Thus, concentrated virus provided a mild transduction effect but exhibited a very toxic effect on fibers and caused dedifferentiation, loss of cross striation, inability to respond to electrical stimulation, and death.

### 3.2. Satellite Cell Isolation and Transduction

**Figure 4:**
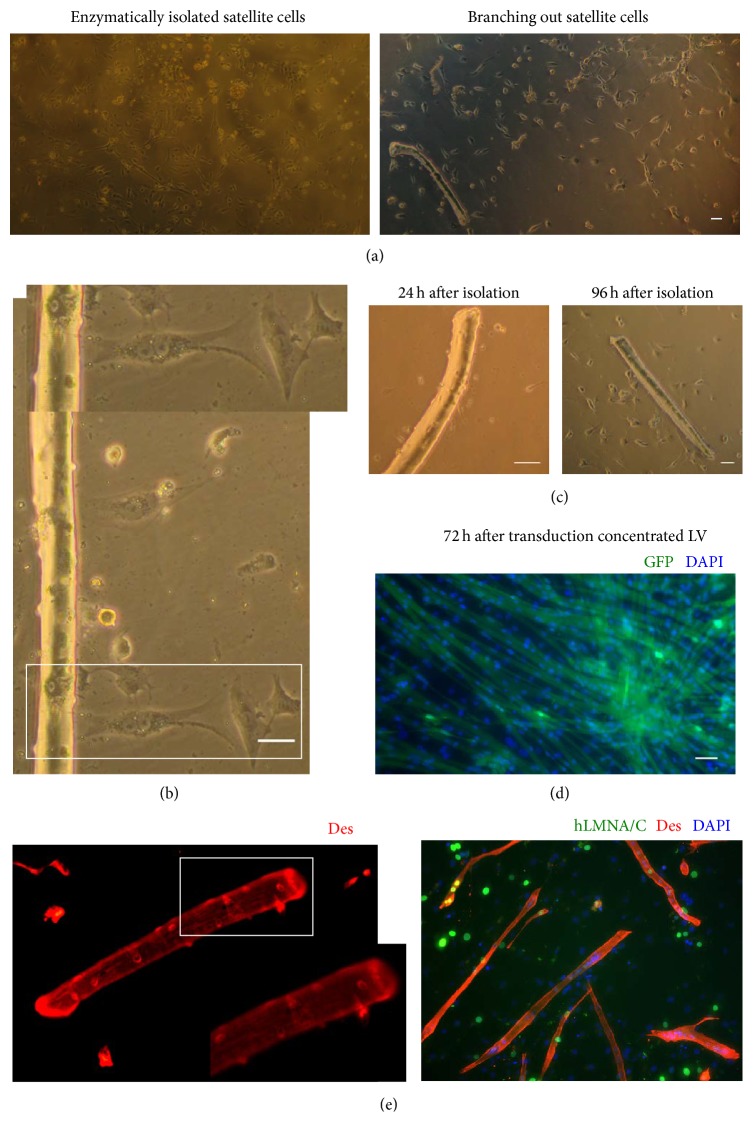
Satellite cell isolation and transduction. (a) 96 hours after isolation enzymatic digestion to obtain a “pure” satellite cell culture resulted in more satellite cells in comparison to experiments where satellite cells were allowed to branch out of muscle fibers. (b) Satellite cell branching out primary muscle fiber, 96 hours after isolation. (c) Muscle fiber and branching out satellite cells 24 hours (left panel) and 96 hours (right panel) after isolation. (d) Enzymatically isolated satellite cells were transduced via concentrated LV (upper panel) encoded GFP. 72 hours after transduction via LV 95% of observed cells express GFP, thus confirming high transduction efficiency (lower panel) encoded human lamin A/C. Myotubes were stained anti-lamin and anti-desmin. Positive staining confirmed myogenicity of transduced cells. Nuclei are shown counterstained with DAPI. Scale bar corresponds to 50 *μ*m (e) Satellite cells branching out primary muscle fiber stained anti-desmin. Positive staining confirms myogenicity of cells located on the muscle fiber surface.

Enzymatic digestion to obtain a “pure” satellite cell culture with satisfactory differentiation capacities resulted in more satellite cells in comparison to experiments where satellite cells were allowed to branch out of muscle fibers maintained in culture for four days. The numbers of enzymatically isolated satellite cells were far greater than in the case of branching out of muscle fibers satellite cells cultivated for similar times (Figure 4(a)). Moreover, enzymatic digestion was more efficient since it was possible to obtain satellite cells from any type of muscle, whereas satellite cells branching out from the muscle fiber were restrained by the numbers of intact fibers isolated. The best results were obtained for the FDB that consists overwhelmingly of short (about 600 *μ*m in length) muscle fibers (Figures 4(b) and 4(c)). Muscle fiber isolation from the soleus muscle often resulted in severe fiber damage and, as a consequence, fewer satellite cells branched out of the surviving fibers.

In satellite cells culture isolated from soleus muscle by enzymatic digestion and transduced by exposure to concentrated LV, 95 ± 3% of the cells (*n* = 5 dishes) expressed GFP 72 h after transduction. The GFP signal remained stable up to and after fourteen days of differentiation when myotubes formation had occurred (Figure 4(d)), confirming the high efficiency and stability of transduction.

We assessed the myogenicity of isolated cells by anti-desmin immunostaining. The number of desmin-positive cells was divided by number of all analyzed cells. The percentage of myogenic cells was 74.3 ± 4.3% (*n* = 450 cells) (Figure 4(e)). Moreover, we estimated the myogenic potential of positively transduced cells. Cells were transduced via LV encoded human lamin and induced to differentiation. Obtained myotubes were stained with anti-lamin and anti-desmin. Positively stained myotubes displayed incorporating high percentage of nuclei expressing human lamin, thus confirming effective transduction of myogenic satellite cells (Figure 4(d), lower panel).

### 3.3. Characterization of Myotubes

**Figure 5:**
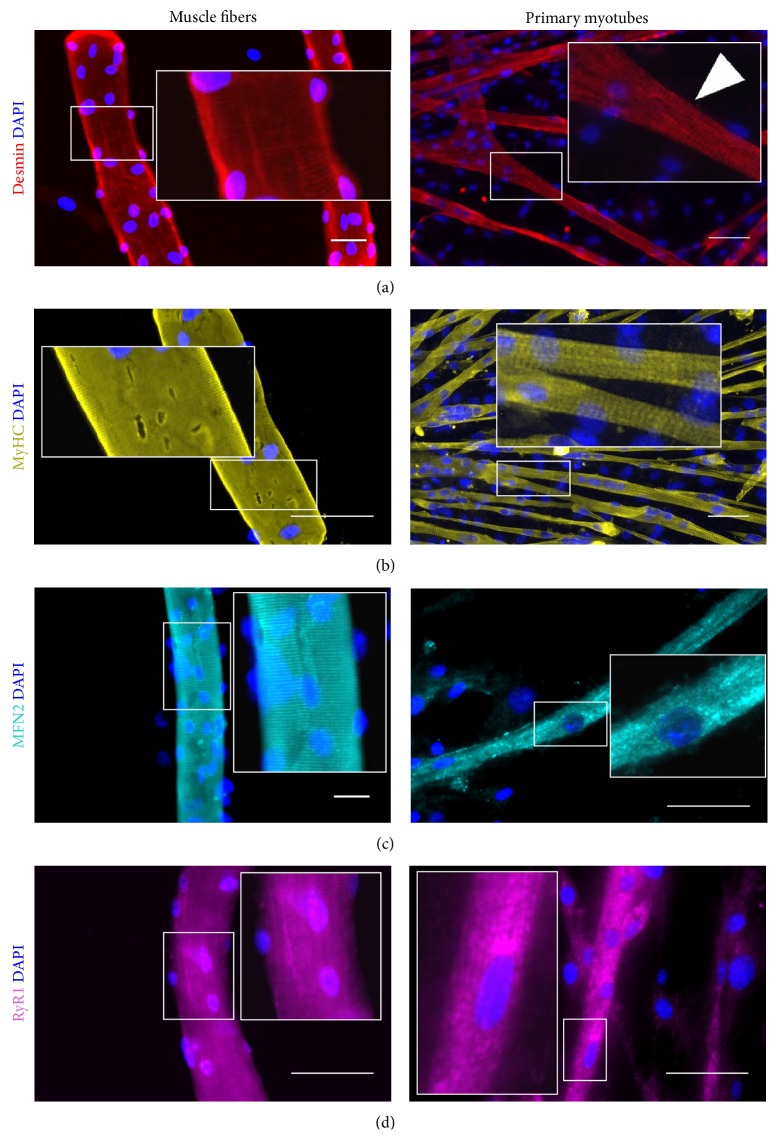
Comparison of muscle fibers and primary myotubes via immunostaining. (a) Anti-desmin, (b) anti-myosin heavy chain, (c) anti-Mitofusin 2, and (d) anti-ryanodine receptor 1. Muscle fibers and primary myotubes show typical cross-striated pattern for desmin and myosin staining. Nuclei were counterstained with DAPI. Scale bar corresponds to 50 *μ*m.

**Figure 6:**
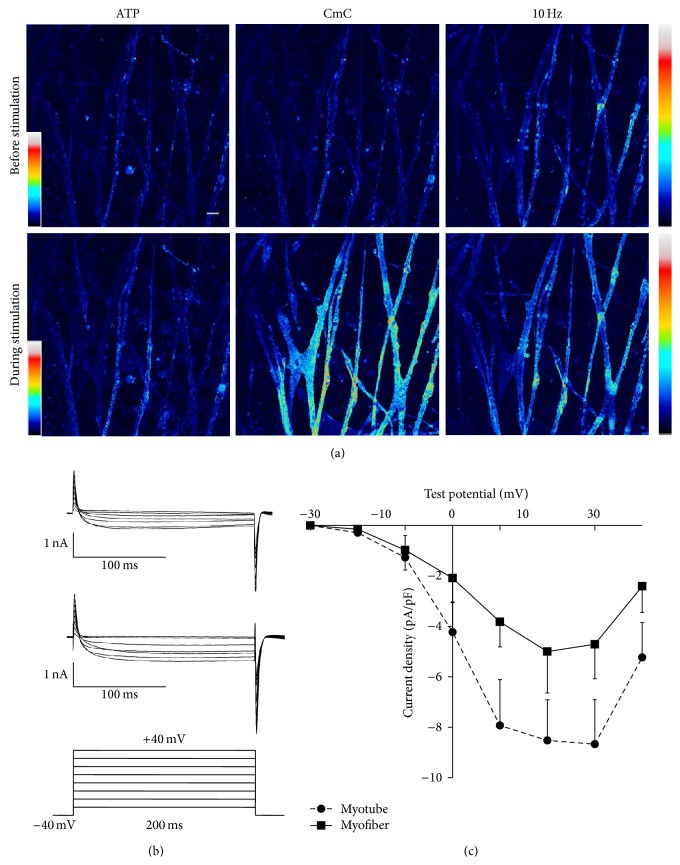
Physiological properties of primary myotubes. (a) Primary myotubes responded to chemical and electrical stimulation within an increase in cytosolic Ca^2+^ followed by mitochondrial Ca^2+^ uptake. Cytosolic Ca^2+^ increase evoked by CmC or electrical stimulations and myotubes responded to stimulations by contraction and [Ca^2+^]_*i*_ increase, confirming developed system of DHPR and RyR. Scale bar corresponds to 50 *μ*m. (b) Representative L-type Ca^2+^ current traces recorded in myotubes after seven days of differentiation (upper panel) and in muscle fibers (middle panel) in response to a series of 200 ms depolarizing steps from −30 to 40 mV in 10 mV increments (lower panel). (c) Current-voltage relationship in adult FDB muscle fibers and myotubes at seventh day of differentiation. Data are presented as mean ± SD (*n* = 3 cells).

For the study of cytosolic and intramitochondrial calcium homeostasis, satellite cells were enzymatically isolated from the slow-twitch soleus muscle. Upon differentiation satellite cells isolated from soleus muscle were able to fuse and form multinucleated myotubes that displayed spontaneous contractions already after 48 h of differentiation (Movie 1 in Supplementary Material available online at http://dx.doi.org/10.1155/2015/594751). Immunocytochemistry confirmed that myotubes expressed proteins typical of late stages of muscle differentiation. Staining for principal sarcomere proteins, desmin and MyHC, gave a cross-striated pattern, similar to that seen in adult muscle fibers (Figures 5(a) and 5(b)). Staining with anti-Mitofusin 2 antibody to visualize mitochondria revealed a patchy staining cross-striated pattern in primary myotubes that was in contrast with the regular cross-striation pattern seen in adult muscle fibers (Figure 5(c)). Ryanodine receptors staining, indicating Ca^2+^ channels in the membrane of the sarcoplasmic reticulum, was abundant in the cytoplasm of myotubes after seven days of differentiation and throughout the myoplasm of muscle fibers (Figure 5(d)). At seven days of differentiation myotubes responded to chemical (2-chloro-*m*-cresol, CmC) and electrical stimulation with release of Ca^2+^ from sarcoplasmic reticulum into the cytosol and subsequent uptake of Ca^2+^ by the mitochondria, confirming the presence of mechanisms of calcium pathways typical for mature muscle (Figure 6(a)). However, the changes in cytosolic Ca^2+^ were not greatly affected by changes in the stimulation frequency from 10 Hz to 100 Hz which is unlike the situation in adult muscle fibers where increasing the frequency causes a marked increase in cytosolic Ca^2+^. The presence of functional dihydropyridine receptors in the membrane of both muscle fibers and myotubes was confirmed by measurement of sarcolemmal Ca^2+^ currents using the patch-clamp. We observed inward current, corresponding with the given experimental conditions to Ca^2+^ current with characteristics typical of those of a L-type Ca^2+^ current in adult muscle fibers (Figures 6(b) and 6(c)). However, the peak current density was significantly smaller (*P* < 0.05) in myotubes than in muscle fibers.

## 4. Discussion

The goal of our study was to identify a robust and relevant cellular model for assessment of intracellular calcium homeostasis in mature muscle cells. We compared primary adult muscle fibers with myotubes formed by satellite cells fusion. We assessed their morphological and physiological properties and checked the ability of cells to undergo LV genetic modification. We demonstrated that primary myotubes formed after satellite cells fusion resembled primary adult muscle fibers in terms of morphology and physiology. Further, primary myotubes, in contrast to muscle fibers, can successfully undergo genetic modification via LV transduction and express the coded proteins in 72 hours after transduction for at least 14 days.

The immunocytochemical data show that primary myotubes expressed myosin and desmin filaments with the typical cross-striated pattern found in adult muscle fibres. Mitofusin 2 was expressed throughout the cytoplasm of myotubes with no apparent cross-striation, indicating that the adult organization of mitochondria had not yet occurred. RyR staining in primary myotubes was found throughout the sarcoplasm indicating an extensive sarcoplasmic reticulum. Primary myotubes were able to contract and to release sarcoplasmic calcium in response to electrical and chemical stimulation indicating a functional excitation-contraction coupling pathway linking L-type channel activation and the RyR in the sarcoplasmic reticulum. Patch-clamp studies showed the presence of Ca^2+^ currents in plasma membrane of primary myotubes although the L-type Ca^2+^ current density was less in myotubes than in adult muscle fibers. Probably due to less number of Ca^2+^ channels per cell in myotubes in comparison with muscle fibers. Previously we showed that sarcoplasmic Ca^2+^ release at 1 Hz stimulation was significantly lower than that at 10 Hz stimulation, while increase of the stimulation frequency to 100 Hz did not result in any further increase in sarcoplasmic Ca^2+^ release [40], unlike the situation in adult muscle fibers where there is increasing [Ca^2+^]_*i*_ with increasing stimulation frequencies [41].

Taken together, the results with primary myotubes are promising because of their morphological and physiological similarity to primary muscle fibers, though they do not completely replicate the situation in adult muscle fibers.

Transduction experiments showed that satellite cells were easy to transduce with LV and were able to retain a GFP signal up to and after formation of myotubes. It is known that primary muscle fibers that are terminally differentiated muscle cells do not easily undergo LV transduction. Indeed in adult fibres 72 hours after transduction little GFP signal was detected but there was a loss of cross-striation pattern and inability to respond to electrical stimulation. It was previously shown that transduction of skeletal muscle fibers was more successful in young (<2 weeks) compared to older (>6 months) mice [31, 42]. High numbers of positively transduced muscle fibers were obtained for adenoviruses and herpes simplex viruses-1 but only in fibers in animals less than two weeks old [31, 42]. Several mechanisms coupled with aging appear to contribute to viral transduction resistance, including downregulation of viral receptors, alteration of basal lamina properties, acquisition of immunological maturity, and decline of satellite cells number [30, 42–44]. Other work suggested that adenoviruses were effective for* in vitro* transduction of FDB muscles. However, the study on FDB muscle did not examine the retention of functional capacity of positively transduced muscle fibers [11]. In our experiments we used adult mice (at least ten weeks old) in order to test and develop a reliable model. We applied LV transduction due to its high transduction titer to genetically modify muscle fibers. However, when a positive signal in the muscle fibers was detected, the muscle fibers displayed a loss of cross-striation and had shortened, probably due to the high toxicity of viral application. On the other hand, LV was able to transduce satellite cells, both those free on the dish and also those attached to the muscle fiber. Moreover, we confirmed that LV efficiently transduced primary myoblasts both at the proliferation (myoblast) stage and at myotubes stage [34, 35]. To sum up while we were unable to achieve positive transduction of primary muscle fibers with retaining their functional activity, we demonstrated that satellite cells were easily transduced by LV and remained physiologically active, in line with previous works [35].

## 5. Conclusions

Muscle dystrophies are accompanied by impairment of intracellular calcium balance. Therefore, it is of particular importance to study calcium pathways within the muscle cells to elucidate precise molecular mechanisms underlying these disorders. Functional analysis of the myotubes formed upon primary satellite cells fusion confirmed their well-differentiated characteristics and their ability to react to electrical and chemical stimulations and the presence of functional L-type Ca^2+^ channels in the plasma membrane. Moreover, unlike adult muscle fibres, satellite cells derived from adult mouse muscle were easily transduced via LV and were able to retain positive signal up to and after formation of myotubes. These results suggest that satellite cells constitute a promising cell model for further experiments aimed at exploring calcium pathways involved in muscle dystrophies caused by mutations in miscellaneous genes.

## Supplementary Material

This movie depicts spontaneously contracting myotubes. Satellite cells were isolated from m. soleus and induced to differentiation for 4 days. Obtained myotubes were amenable to contraction without extra stimuli. Multiple foci of contraction can be observed.

## Abbreviations

Adeno: Adenovirus
AAV: Adenoassociated virus
CmC: 2-Chloro-*m*-cresol
DMD: Duchene muscular dystrophy
FDB: Muscle flexor digitorum brevis
GFP: Green fluorescent protein
HSV: Herpes simplex virus
LV: Lentivirus
mdx: Transgenic mouse strain carried mutant dystrophin gene
MOI: Multiplicity of infection
RyR: Ryanodine receptor
SR: Sarcoplasmic reticulum.
